# Porcine Deltacoronavirus in Mainland China

**DOI:** 10.3201/eid2112.150283

**Published:** 2015-12

**Authors:** Nan Dong, Liurong Fang, Songlin Zeng, Qianqian Sun, Huanchun Chen, Shaobo Xiao

**Affiliations:** Huazhong Agricultural University, Wuhan, China (N. Dong, L. Fang, S. Zeng, Q. Sun, H. Chen, S. Xiao);; The Cooperative Innovation Center for Sustainable Pig Production, Wuhan (L. Fang, H. Chen, S. Xiao)

**Keywords:** porcine, deltacoronavirus, mainland China, viruses, pigs, swine, piglets

**To the Editor:** Porcine deltacoronavirus (PDCoV) was discovered in 2012, during a study to identify new coronaviruses in mammals and birds in Hong Kong (*1*). In February 2014, this novel porcine coronavirus was detected in pigs in Ohio, United States (*2*), and has since been reported in at least 17 US states (*3*–*5*). Concern regarding the epidemiology, evolution, and pathogenicity of this emerging virus is increasing. Recently, PDCoV was identified in South Korea (*6*). We report PDCoV in mainland China. 

Since December 2010, a large-scale outbreak of diarrhea in suckling piglets has occurred on swine farms in mainland China (*7*). The causative agent was considered to be a variant of porcine epidemic diarrhea virus (PEDV) (*8*), and the role of PDCoV in the outbreak was not investigated at that time.

Using 2 pairs of specific primers to detect PDCoV, as described by Wang et al. (*2*), we tested 215 intestinal or fecal samples collected at various times during 2004–2014 from piglets with clinical diarrhea in Anhui, Guangxi, Hubei, and Jiangsu provinces, mainland China (Technical Appendix Table 1). All samples were submitted from commercial pig farms to our laboratory for enteropathogen detection. Of these samples, 165 (124 from Hubei, 41 from Jiangsu) had been collected in 2014, and 50 (40 from Jiangsu, 6 from Anhui, 4 from Guangxi) had been collected during 2004–2013 and preserved in our laboratory. The 215 samples were simultaneously tested for PEDV and transmissible gastroenteritis virus (TGEV) by using the primers listed in Technical Appendix Table 2. Of the samples tested, 14 (6.51%) were positive for PDCoV, 110 (51.2%) were positive for PEDV, and 5 (2.3%) were positive for TGEV. Of the 14 PDCoV-positive samples, 7 (50%) were also positive for PEDV; 2 of the 215 samples were co-infected with PEDV, TGEV, and PDCoV (Technical Appendix Table 1). Previous studies have shown that prevalence of PDCoV in the midwestern United States in 2014 was high (30%) and that PDCoV co-infections with other pathogens (such as PEDV and rotavirus) are more common (78% of PDCoV infections) (*4*). At the same time in mainland China, the rate of PDCoV positivity was lower (7.27%), whereas that of PEDV was higher (52.73%), suggesting that PEDV remains the main causative agent of piglet diarrhea diseases in mainland China. Similarly, in South Korea in 2014, only 2 PDCoV-positive samples were detected in 113 samples of diarrhea from pigs (*6*).

We also examined the collection dates and geographic locations of the PDCoV-positive samples and found that PDCoV was detected in pigs in Hubei (8/124), Jiangsu (4/81), and Anhui (2/6) Provinces. However, all samples from pigs in Guangxi Province were negative for PDCoV. All PDCoV-positive samples from Hubei and Jiangsu Provinces had been collected in 2014, whereas the 2 PDCoV-positive samples from Anhui Province had been collected in 2004.

Among the PDCoV-positive samples, we selected 3 for complete genome sequencing with 16 pairs of overlapping primers, as described previously (*2*): one (CHN-AH-2004) collected from Anhui Province in 2004, one (CHN-HB-2014) from Hubei Province in 2014, and one (CHN-JS-2014) from Jiangsu Province in 2014. These complete genome sequences have been deposited in GenBank under accession nos. KP757890 (CHN-AH-2004), KP757891 (CHN-HB-2014), and KP757892 (CHN-JS-2014). The complete genome sequences of 3 PDCoV strains from pigs in mainland China shared high nucleotide identities (>98.9%) with all previously reported PDCoV strains. Previous studies found that Hong Kong strain HKU 15–44 and all PDCoV strains from the United States and South Korea have a 3-nt insertion in the spike gene, which is not present in Hong Kong strain HKU 15-155 (*2*–*6*). This insertion is also present in CHN-AH-2004, whereas CHN-HB-2014 and CHN-JS-2014, like HKU 15-155, lack this insertion (online Technical Appendix Figure).

Although all reported PDCoV strains from China shared high similarity with each other, a phylogenetic tree based on all available complete PDCoV genome sequences showed that these PDCoV strains clearly cluster in different clades (Figure). Strain CHN-JS-2014 shares an ancestor with the strains from the United States and South Korea. CHN-AH-2004 and HKU15-44 share a common ancestor, and CHN-HB-2014 shares a common ancestor with CHN-AH-2004 and HKU15-44.

**Figure:**
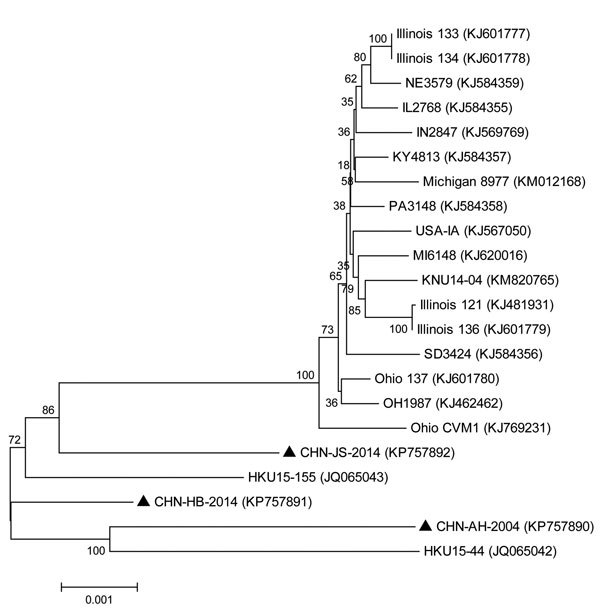
Phylogenetic tree of all complete porcine deltacoronavirus genome sequences available in February 2015. The phylogenetic tree was constructed by using the distance-based neighbor-joining method in MEGA 6.06 software (http://www.megasoftware.net/). Bootstrap values were calculated with 1,000 replicates. The number on each branch indicates bootstrap values. The reference sequences obtained from GenBank are indicated by strain abbreviations and GenBank accession numbers. Triangles indicate the 3 strains from mainland China. Scale bar indicates nucleotide substitutions per site.

As an emerging virus, PDCoV has been poorly understood. Our data suggest that PDCoV has existed in mainland China for at least 11 years. Although the rate of PDCoV infection detected in mainland China in this study was relatively low, the results may not accurately reflect the prevalence of PDCoV in mainland China because the tested samples were collected from only 4 provinces. Extensive surveillance is required to define the epidemiology and evolution of PDCoV in mainland China. Recent confirmation that PDCoV is enteropathogenic in gnotobiotic pigs (*9*) highlights the need for effective vaccines against this emerging virus.

Technical Appendix Additional methods used to detect porcine deltacoronavirus in pigs from mainland China and virus sequencing results.
