# Supplementary material for: Porcine Deltacoronavirus in Mainland China
Source: Emerg Infect Dis. 2015 Dec;21(12):2254–5. doi: 10.3201/eid2112.150283 (PMC4672429; doi:10.3201/eid2112.150283)
Supplement: Technical Appendix — Additional methods used to detect porcine deltacoronavirus in pigs from mainland China and virus sequencing results. [file 15-0283-Techapp-s1.pdf]

Article DOI: http://dx.doi.org/10.3201/eid2112.150283

Porcine Deltacoronavirus in Mainland China

Technical Appendix

Technical Appendix Table 1. Detection of porcine deltacoronavirus (PDCoV), porcine epidemic diarrhea virus (PEDV), transmissible gastroenteritis virus (TGEV) in samples from different provinces of mainland China*

	No. samples positive /total no. samples tested		
Province	PDCoV	PEDV	TGEV
Hubei	8/124	63/124	0/124
Jiangsu	4/81	38/81	2/81
Anhui	2/6	6/6	3/6
Guangxi	0/4	3/4	0/4
All provinces	14/215	110/215	5/215

*A total of 215 samples from 4 provinces of mainland China were detected for PDCoV, PEDV, TGEV, and 165 samples were collected in 2014, 50 were previous samples preserved in our laboratory dated from 2004-2013.

Technical Appendix Table 2. Primers used for detection of porcine epidemic diarrhea virus (PEDV) and transmissible gastroenteritis virus (TGEV).

Primer identification	Primer sequence (5’- 3’)	Size (bps)
PEDV-F	TCTGCGTTCTTGTATGGTGTC	595
PEDV-R	TCCTGAAAACTGACAGAAGCC	
TGEV-F	AGATGGAGTTGTCTGGGTT	739
TGEV-R	ATCTTGCTCTGACCTTTCT	

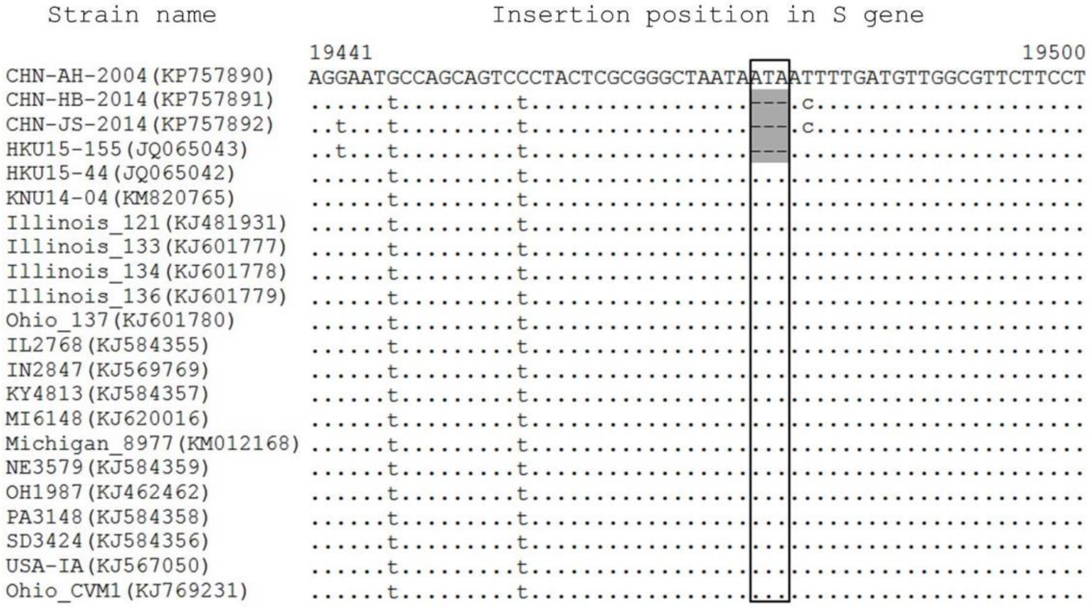

Technical Appendix Figure. Alignment of partial sequences of the spike gene of porcine deltacoronavirus (PDCoV). A dot indicates that the nucleotide exactly matches the consensus. A dash indicates that the nucleotide deletes compared to the reference sequence. The position for the 3-nt insertion is highlighted by a box.
